# Diversity and Distribution Patterns of Endemic Medicinal and Aromatic Plants of Iran: Implications for Conservation and Habitat Management

**DOI:** 10.3390/ijerph19031552

**Published:** 2022-01-29

**Authors:** Mohammad Bagher Hassanpouraghdam, Hamideh Ghorbani, Marzieh Esmaeilpour, Mac H. Alford, Maciej Strzemski, Sławomir Dresler

**Affiliations:** 1 Department of Horticultural Science, Faculty of Agriculture, University of Maragheh, Maragheh 55181-83111, Iran; h_ghorbani30@yahoo.com; 2 Department of Geography, University of Maragheh, Maragheh 55181-83111, Iran; m.esmaeilpour@maragheh.ac.ir; 3 School of Biological, Environmental, and Earth Sciences, University of Southern Mississippi, Hattiesburg, MS 39406, USA; mac.alford@usm.edu; 4 Department of Analytical Chemistry, Medical University of Lublin, 20-093 Lublin, Poland; maciej.strzemski@poczta.onet.pl (M.S.); slawomir.dresler@umlub.pl (S.D.); 5 Department of Plant Physiology and Biophysics, Institute of Biological Science, Maria Curie-Skłodowska University, Akademicka 19, 20-033 Lublin, Poland

**Keywords:** biodiversity hotspots (BHs), Irano-Anatolian Hotspot, medicinal and aromatic plants (MAPs), endemism, extinction, conservation

## Abstract

Iran, with its unique climatic and topographic conditions, is home to about 8200 species of vascular plants. Approximately 2300 of the 8200 species are popularly characterized as medicinal or aromatic. Here, we compile information about the endemic medicinal and aromatic plants (MAPs) of Iran and map their distributions. Our survey found 180 endemic species of MAPs, belonging to 10 families and 30 genera. The majority of species are found in Lamiaceae, Fabaceae, and Apiaceae, with 86, 30, and 18 species, respectively. Approximately 70% of these plants have been recorded in the 10 provinces of Esfahan, Kerman, Fars, Tehran, Chaharmahal va Bakhtiari, East Azarbaijan, Lorestan, West Azarbaijan, Hamadan, and Mazandaran. These provinces are located in the Iran-o-Turanian region, one of the three major phytogeographic regions in Iran, which covers five areas of endemism (i.e., Azarbaijan, Zagros, Kopet Dagh-Khorassan, Alborz, and Central Alborz). So, Iran-o-Turanian region is the main center of diversity for the Iranian endemic MAPs. The north, center and western parts of Iran are rich in MAPs and could be considered as the dominant biodiversity hotspots of Iran more seemingly due to the diverse climatic and geographic assortment which generates the highest frequency and distribution of MAPs. Many of these MAPs are at the edge of extinction due to the unwise, unscientific harvesting and/or global climate change. Therefore, there is an urgent need to conserve and propagate some of these important MAPs to save them from extinction and also to ensure the availability of raw materials for their use and future research into their efficacy. Furthermore, identifying the areas of endemism (AEs) is an essential part of ongoing regional conservation management programs in Iran and worldwide.

## 1. Introduction

Endemic plant species are plants that exist in one particular geographical region and nowhere else on the globe [1], and endemism is the status of being endemic or being restricted to a distinct geographical district [2]. The geographical region can be as small as an island or as large as a continent. Areas with high concentrations of endemic species and with significant habitat loss are also referred to as “Biodiversity Hotspots” (BHs) [3]. Currently, 36 areas around the world are considered BHs [2].

The fundamental hazard of endemic species is that they are more susceptible to extinction. Since they are limited geographically, the devastation of their habitat due to man-made enterprises and/or global climate change could reduce their populations drastically [4]. Because endemism is deemed as a substantial factor for biodiversity conservation at the local, national, and global scale, identifying the number and distribution of endemic plants in a biogeographic area is a preliminary point for evaluating the protection of that defined region [5].

Of the 422,000 species of vascular plants in the world, 50,000–80,000 of them are used medicinally and contain valuable ethnobotanical and remedial information that could navigate new drug exploration [6,7]. As one of the significant bio-resource centers of the world, the Asian continent accounts for over 38,660 species of MAPs (Medicinal and Aromatic Plants) [8]. Nowadays, the application of MAPs is increasing due to their rich capacity for the treatment of diverse maladies and their fewer side effects [9]. Therefore, the tendency in worldwide research has focused more on the search for new medicines and active compounds of MAPs rather than on the cultivation and/or domestication of the plant species with this characteristic potential [10]. Increasing population pressure, overharvesting, unscientific collection by untrained persons, excessive grazing, fire, and global climate change have placed many of these plants at the risk of extinction [11]. Experts estimate that the Earth is losing at least one potential primary drug every two years [6].

Setting priorities is necessary for the conservation management of MAPs. So, identifying BHs in the world and mapping the AEs (Areas of Endemism)of every country can be used as a powerful method for the prioritization of the endemic MAPs conservation [3]. A total of 36 BHs have been designated, covering 16.7% of Earth’s land surface and home to 77% of all endemic plant species [2]. For conservation management purposes, however, they are practically too large, so an appropriate method to achieve protection of these species is the identification and studying of the AEs within a global biodiversity hotspot [1,12].

One of the global BHs located entirely within southwest Asia is the Irano-Anatolian hotspot, which extends over an area of about 899,773 km^2^ and is home to about 6000 plant species [13]. Iran covers an estimated 54% of the surface area of the Irano-Anatolian hotspot [14]. Another BH, the Caucasus, includes Georgia, Azarbaijan, and a small portion of northern Iran (around 10%) [1,12]. So, Iran is home to two of the world’s BHs: The Irano-Anatolian and Caucasus.

Iran is a vast country (1,648,195 km^2^), with different climates ranging from mainly arid to semi-arid and also mountainous [15], and is at the intersection of three well-known phytogeographic areas (the Iran-o-Turanian, the Saharo-Sindian, and the Euro-Siberian) [16]. The majority of Iran is located in the Iran-o-Turanian region and is divided into two sub-regions: mountainous areas and an area of high plains and deserts [15]. The driest portion of Iran-o-Turanian (desert sub-region) is dominated by arid and hyper-arid climates and has reasonable plant diversity.

It seems that the environmental and climatic features have had a fundamental influence on the endemic diversity and richness of Iran. The dominant climates of Iran include arid, semi-arid, hyper-arid as well as small territories of humid, semi-humid, highly-humid, and Mediterranean [17].

According to Noroozi et al. (2019), the Iran-o-Turanian region harbors about 88% of the Iranian endemic species. Given the climatic conditions and diversity of endemic species, this region has been further subdivided into five AEs: Alborz, Central Alborz, Zagros, Azarbaijan, and Kopet Dagh-Khorasan [1,12,14].

The Alborz and Zagros Mountains expand in a northwest-northeast and northwest-southeast orientation [18] and are situated in East and West Azarbaijan, Tehran, Kurdistan, Kermanshah, Lorestan, Fars, Chaharmahal va Bakhtiari, Esfahan, Yasouj, Markazi, and the northern part of Khozestan Provinces. Other significant AEs are Iranian Azarbaijan in the northwest and Kopet Dagh-Khorasan that stretches from the eastern boundary of the Caspian Sea into northeastern Iran [19].

The spectacular nature of this Iranian plateau with five AEs, is full of several species of endemic MAPs with important potential pharmaceutical and therapeutic properties that might not have been widely reported or thoroughly studied to date. People have long occupied this area, with an early center of civilization that dates back to the Babylonian-Assyrian era [20]. One of the most considerable parts of this ancient heritage is the science of people who figured out helpful plants for health improvement, with subsequent generations appending their own experience and skill to this custom [20].

Approximately 8200 vascular plant species are recognized in Iran, of which almost 2300 are medicinal and aromatic (28%) [13,21,22]. Among these are numerous endemic species that are only known from Iran. Many studies have been performed on species of endemic MAPs based on chemical composition and biological activity, but little attention has been paid to the distribution patterns, areas of endemism, and conservation zones of endemic MAPs in Iran.

In this review of the endemic MAPs of Iran, we compiled and analyzed data with the following aims: (1) to determine the families and genera with high endemic richness, (2) to determine the provinces with higher endemic species richness, (3) to recognize the distribution patterns and areas of endemism for medicinal and aromatic species of the country, and (4) to present some suggestions for conservation of these areas of endemism.

## 2. Methods

We reviewed scientific studies published in reliable journals and books. Pertinent literature was searched for in electronic databases such as Magiran, Scopus, Google Scholar, Web of Science, Science Direct, and PubMed and books (including some dictionaries of Iranian plants compiled by Valiallah Mozafarian and Ahmad Ghahreman [23], and the Red Data Book of Iran: A Preliminary Survey of Endemic, Rare & Endangered Plant Species in Iran, compiled by Adel Jalili and Ziba Jamzad) [24], using specific search terms such as biodiversity hotspots, Irano-Anatolian hotspot, medicinal and aromatic plants, medicinal herbs, traditional plants, endemism, extinction, and conservation.

We do not claim to have included every source about endemic MAPs of Iran, rather we focused on data available on the internet and in libraries that are accessible to scholars. We found and reviewed a total of 165 articles that provided details about endemic Iranian MAPs that are used to treat various ailments and disorders.

From these resources, we compiled a list of endemic MAPs, showing family, scientific names, common names, parts used, therapeutic effects and ethnopharmacological properties, provinces (habitats), and references for each species. 

The distribution maps were prepared using a geographic information system (ArcGis 10.2.2), with the country was divided into three major phytogeographic districts (the Iran-o-Turanian, the Saharo-Sindian, and the Euro-Siberian) and five AEs (Alborz, Central Alborz, Zagros, Azarbaijan, and Kopet Dagh-Khorasan) according to the distribution of the endemic MAPs across provinces of Iran. A distribution map of endemic MAPs by different climatic conditions of Iran was also drawn.

## 3. Results and Discussion

### 3.1. Taxonomic Divisions of Endemic MAPs in Iran

**Figure 1:**
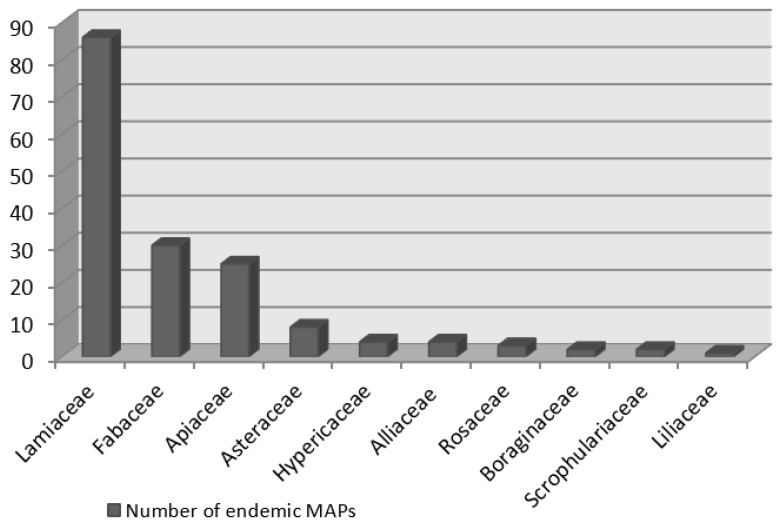
The species number of the ten most endemic-rich families of Iranian medicinal and aromatic plants.

**Figure 2:**
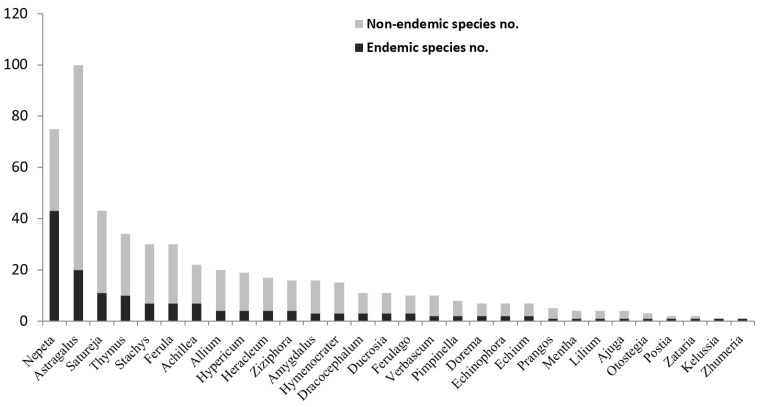
The frequency of endemic and non-endemic species of the 30 most endemic-rich genera of the Iranian medicinal and aromatic plants.

Vascular plants of Iran comprise 65 families, 359 genera, and about 8200 species, of which 2597 of them are endemic (32% of all native species) [1,12]. Of these, we found that 180 endemic MAPs, which belong to 30 genera and 10 families, are used in Iran. The 10 families in terms of the number of endemic MAPs are Lamiaceae (84 spp.), Apiaceae (37 spp.), Fabaceae (21 spp.), Asteraceae (7 spp.), Hypericaceae (2 spp.), Alliaceae (2 spp.), Rosaceae (3 spp.), Boraginaceae (3 spp.), Scrophulariaceae (3 spp.), and Liliaceae (3 spp.) (Figure 1). A total of 10 families are representing the major medicinal plants of Iran with about 165 most dominant species (Figure 1 and Figure 2 and Table 1).

The largest number of Iranian endemic MAPs is observed in Lamiaceae and Fabaceae, with 86 and 30 species, respectively (Figure 1). The dominant MAPs genera of Iranian flora are presented in Figure 2 from which the notable genera include *Nepeta* from Lamiaceae and *Astragalus* from Fabaceae.

Lamiaceae is the family with the largest number of species in terms of endemic MAPs (12 genera and 86 species, Table 1), which is due to the hyper-diverse genus *Nepeta* with 43 species (53% endemic, Figure 2). The genus *Nepeta* consists of about 300 species widely distributed in Europe, Asia, and some areas of Africa. Iran is one of the primary centers of this genus, with 79 species [25]. The genus *Nepeta*, with the common Persian name of “Pune-sa”, is widely used in the folk medicine of Iran for diuretic, digestive, diaphoretic, antitussive, antispasmodic, anti-asthmatic, febrifuge, emmenagogue, and sedative effects [25,26].

The second most prominent family in terms of endemic MAPs is Fabaceae (Figure 1), with most species in the genus *Astragalus*. Iran is one of the world’s centers of *Astragalus* diversity, which makes up 21% of Iran’s endemic vascular plants. Furthermore, Fabaceae is the second largest family of Iranian flora containing a large number of native species (850 species) and the total number of endemic species (527 species), but we found only 30 endemic MAPs (Figure 2, Table 1). Meanwhile, the interest in the chemical compounds of different species of *Astragalus* has been increasing; these data may change as the new endemic MAPs species continue to be characterized [15].

Other families with high numbers of endemic MAPs are Apiaceae, Asteraceae, Rosaceae, Boraginaceae, Hypericaceae, Liliaceae, Scrophulariaceae, and Alliaceae. As species conservation and taxonomy are often assumed to be interdependent activities, it is suggested that these families should be further studied, especially their medicinal and aromatic species, since a shortage of taxonomic information can cause problems for conservationists [13].

In addition to the traditional and newly characterized medicinal plants, there is another group, namely the potential medicinal plants. These kinds of plants are species that are thought to contain bioactive compounds that are medicinally efficacious. They have not been medically proven, but they are used as ingredients for traditional medicine. Table 2 presents 80 endemic MAPs of Iran which have only been explored superficially, despite having beneficiary effects. The highlighted species need to be more thoroughly studied for their active ingredients to justify the traditional usage of the species and to aid the search for components that may be used in modern medicine [27,28,29,30].

**Table 1:** List of endemic medicinal and aromatic plants identified from Iran.

Scientific Name	Common Name (Vernacular Name)	Parts Used	Therapeutic Effects & Ethno Pharmacological Properties (The Number Inside Paranthesis of Families Refers to the Number of Endemic Species)	Province(s)	Ref.
Alliaceae (2)					
*Allium hirtifolium* Boiss.	Mosir	bulb	antioxidant, hypertension, rheumatoid, inflammation, wounds healing, antibacterial, antifungal, and anticancer	West Azarbaijan, Kurdestan, Kermanshah, Hamadan, Lorestan, Esfahan, Chaharmahal va Bakhtiari, Kohkiluyeh va Boyer-Ahmad, Fars, Arak, Yasouj,	[31,32]
*Allium jesdianum* Boiss. & Buhse	Bon-e-Sorkh or Lizak	aerial parts	antifungal exhibits cytostatic and cytotoxic activities against several malignant tumor cells	Chaharmahal va Bakhtiari, Lorestan	[33]
Apiaceae (18)					
*Dorema ammoniacum* D. Don.	Kandal, Vasha and Koma-kandal	ripe fruit, stem, leaf root, and flower	carminative, diaphoretic, mild diuretic, expectorant, antimicrobial, and vasodilator agent	Yazd, Esfahan, Semnan, Fars, Sistan va Baluchestan, Tehran	[34]
*Dorema aucheri* Boiss.	Kandal-e Kohi	ripe fruit, stem, leaf root, and flower	lowering blood pressure, liver injury stimulant, antispasmodic, expectorant, chronic bronchitis, asthma, and anti-oxidative	Hamadan, Kermanshah, Lorestan, Esfahan, Fars, Kerman, Semnan	[35]
*Ducrosiaanethifolia* (de Candolle.) Boiss.	Moshgak and Moshkbu	aerial parts	cold remedy, cures stomach hurting, sedative and painkiller activity, anti-headache, back pain, colic, and colds, effective against insomnia and anxiety	Fars	[36,37,38]
*Ducrosia assadii* Alava.	Moshgake bakraei	aerial parts	anti-inflammatory, antiseptic, carminative, and soporific,	Kerman	[39]
*Ducrosia flabellifolia* Boiss.	Moshgake badbezani	aerial parts	antioxidant	Kerman	[36,40]
*Echinophora cinerea* (Boiss.) Hedge & Lamond.	Khosharizeh Kohestani	aerial parts	antioxidant and antidiabetic	Hamadan, Lorestan, Kohkilouyeh va Boyer-Ahmad, Fars, Chaharmahal va Bakhtiari	[41,42]
*Echinophora platyloba* de Candolle.	Khosharizeh	aerial parts	antimicrobial, antioxidant	East and West Azarbaijan, Kurdestan, Hamadan, Lorestan, Arak, Esfahan, Fars, Kerman, Khorasan, Tehran	[41,43,44,45]
*Ferula assa-foetida* Linnaeus.	Anghoseh	gum, resin	antispasmodic, aromatic, carminative, digestive, expectorant, laxative, sedative, analgesic, anthelmintic, aphrodisiac, anticonvulsant, diuretic, tonic, and antiseptic	Kerman, Khorasan	[46,47]
*Ferula persica* Boiss.	Koma	aerial parts, root	anti-pigmentation in Serratia marcescens, cytotoxic, antibacterial, anti-fungal, cancer chemo-preventive, reversal of multi-drug resistance, anti-inflammatory, lipoxygenase inhibitory activity, laxative, carminative, antihysteric; treatment of lumbago, diabetes, rheumatism, and backache	Tehran,	[46,47,48,49]
*Ferula flabelliloba* Rech. f. & Aell.	Koma-e Binaloodi	aerial parts, root	expectorant, aphrodisiac, sedative, antiseptic, carminative, antibacterial, laxative, analgesic, anthelmintic, diuretic	Khorasan	[46]
*Ferulago carduchorum* Boiss and Hausskn.	No known common name	root	antibacterial activity	Illam, Kerman	[50,51]
*Ferulago contracta* Boiss. & Hausskn.	Chavil-e Khoshei	aerial parts	sedative, tonic, digestive, aphrodisiac, and in the treatment of intestinal worms and hemorrhoids	Yazd	[52]
*Heracleum anisactis* Boiss.	Golpar Damavandi	root, stem, leaf, and fruit	antiseptic, carminative, digestive, and analgesic	Ardabil	[53,54]
*Heracleum gorganicum* Rech. F.	Golpar Gorgani	root, stem, leaf, and fruit	carminative, antiseptic, digestive, epilepsy, and analgesic	Golestan	[53,55]
*Heracleum rechingeri* Manden.	Golpar Asalemi	root, stem, leaf, and fruit	carminative, antiseptic, anthelminthic, diuretic, digestive, and analgesic	Mazandaran	[53,56]
*Kelussia odoratissima* Mozaff.	Keluss or Karafs-e- Bakhtiari	leaf, stem, seed, and root	anti-inflammatory, anti-viral, anti-diabetic, anti-cancer, anti-stress, antioxidant, antihyperlipidemic, ulcerative colitis, sedative, antibacterial, pulmonary hypertension, and anti-tumor	Chaharmahal va Bakhtiari, Esfahan	[57,58]
*Pimpinella deverroids* Boiss.	Jafari kohi or Anison	fruit	antioxidant	Kermanshah, Hamadan, Lorestan, Esfahan, Yazd, Fars, Chaharmahal va Bakhtiari	[59]
*Prangos cheilanthifolia* Boiss.	Joshire Azarbaijani	aerial parts	emollient, carminative, antifungal, antioxidant, antibacterial, anti-HIV, tonic, antiflatulent, anthelmintic	East and West Azarbaijan, Esfahan, Yazd, Kerman, Tehran	[60]
Asteraceae (7)					
*Achillea aucheri* Boiss.	Boomadaran Damavandi	aerial parts with flowering tops	antispasmodic, anti-inflammatory, diuretic, and diaphoretic, treatment of hemorrhage, pneumonia, rheumatic pain, and wounds	East and West Azarbaijan, Tehran	[61,62,63]
*Achillea biebersteinii* Afan.	Bumadaran-e Zard	aerial parts with flowering tops	hypoglycemic, nerve tonic, anti-hemorrhoid, antidiarrhea, antacid, carminative, appetizer, anthelmintic and antibacterial	Hamadan, East-Azarbaijan	[63,64,65]
*Achilleaeriophora* de Candolle.	Bumadaran-e Shirazi	aerial parts with flowering tops	feverish conditions, common cold, digestive complaints, slow healing wounds, and skin inflammations	Sistan va Baluchestan, Fars, Hormozgan, Khuzestan, Kerman	[63,64,66,67]
*Achillea kellalensis* Boiss. & Hausskn.	Bumadaran-e Bakhtiari Golberrenjas Bumadaran-e-Sabzekoh	aerial parts with flowering tops	remedy for edema, burns, wounds, carminative, indigestion, skin infection, gastric ulcer, anti-bacterial, hemorrhage, dysmenorrhoea, enema, and diarrhea	Lorestan, Esfahan, Chaharmahal va Bakhtiari, Fars	[63]
*Achillea oxyodonta* Boiss.	Bumadaran-e Shemirani	aerial parts with flowering tops	spasmolytic, choleretic, treatment of wounds, and anti-inflammatory activities	Tehran, Hamadan, Esfahan	[63,68]
*Achilleatalagonica* Boiss.	Bumadaran-e Taleghani	aerial parts with flowering tops	fever, asthma, skin inflammation, jaundice, and liver ailments.	Chaharmahal va Bakhtiari, Tehran, Khuzestan, East and West Azarbaijan, Kurdestan, Lorestan, Esfahan	[63,69,70]
*Postia puberula* Boiss. & Blanche	No known common name	aerial parts	antioxidant activity	Lorestan	[71]
Boraginaceae (2)					
*Echium amoenum* Fisch. & C.A. Mey.	Gol-e-Gavzaban Irani	petal	tonic, tranquilizer, diaphoretic, cough suppressant, and a remedy for sore throat and pneumonia,	Golestan, East and west Azarbaijan, Mazandaran, Gilan, Hamadan	[72,73]
*Echium khuzistanicum* Mozaffarian.	Gol-e-Gavzaban Khuzestani	petal	anti-allergic, antibacterial, antiviral, antifungal, antioxidant, anti-inflammatory, and wound healing	Khuzestan	[74]
Fabaceae (8)					
*Astragalus adscendens* Boiss. & Hausskn.	Gaz-e Khansar Gavan-e Gaz- Angabin	stem, leaf, flower, root, manna	antioxidant, laxative, antispasmodic, antiheadache, antidiabetic, febrifuge, and digestive	Esfahan, Chaharmahal va Bakhtiari, Lorestan and Khuzestan	[75,76]
*Astragalusfasciculifolius* Boiss.	Anzrot Gonjed	leaf, flower, root, stem, seed	tightening the roots of teeth, cough, nutritious, kidney, stomach ache, chest infection, toothache treating heart disease and cancer	Sistan va Baluchestan	[76]
*Astragalusgossypinus* Fisch.	Gavan-e Panbei	gum	cough, anti-fungal, skin diseases, hair gel	Kermanshah	[76]
*Astragalus hamosus* Linnaeus.	Iklil-ul-Malik Nakhonak	pod	headache, vertigo, strokes and dementia gastrointestinal upset, inflammations, respiratory discomfort, and urinary complications	Esfahan, Fars and Bushehr	[77]
*Astragalus fischeri* Buhse ex Fisch.	Shoun korouchok	aerial parts, seed, root	toothache, backache, bone ache, kidney ache, bone fracture, diabetes, and to induce abortion	Esfahan, Fars and Bushehr	[76]
*Astragalusmicrocephalus* Willd.	Kalelak-	stem, root	asthma, strengthen hair	Tehran, Mazandaran	[76]
*Astragalus Chrysostachys* Boiss.	Gavan	root	antioxidant and antibacterial	East Azarbaijan	[78]
*AstragalusPodolobus* Boiss. & Hohen.	Katek	aerial parts, leaf, flower, seed	anemia	Hormozgan	[79]
Hypericaceae (2)					
*Hypericum asperulum* Jaub. & Spach.	Gol-e Raei Lorestani	flowering aerial parts	anti-depression, sedative, strengthens the nervous system and antioxidant	Kurdestan	[80]
*Hypericum dogonbadanicum* Assadi.	Hofariqun	flowering aerial parts	anti-depression, sedative, strengthens the nervous system and antioxidant	Kuhkiluye va Boyer-Ahmad	[80,81]
Lamiaceae (55)					
*Ajuga chamaecistus* Ging.	Labdisi	aerial parts	hypoglycemic, anti-inflammatory, analgesic, anti-arthritis, antipyretic, hepatoprotective, antibacterial, antifungal, antioxidant, cardiotonic, and antimalarial	Tehran, Semnan, East and West Azarbaijan, Hamadan, Kermanshah, Arak, Esfahan	[82,83]
*Dracocephalum kotschyi* Boiss.	Badranjboye Denayi	aerial parts	antioxidant, antibacterial, anticancerous, antinociceptive, antihyperlipidemic, antispasmodic, cytotoxic, and immunomodulatory effects	Esfahan, Yasuj, Mazandaran, Tabriz, Golestan, Hamadan, Fars, Semnan, Tehran	[84,85,86]
*Dracocephalum polychaetum* Linnaeus.	Badranjboye Kermani	aerial parts	anti-depression, anticancer, antimicrobial, and vasodilative effects	Kerman	[87,88]
*Dracocephalum surmandinum* Rech. f.	Badranjboye Sormandi	aerial parts	tonic, and gastrointestinal disorders	Esfahan, Chaharmahal va Bakhtiari	[89]
*Hymenocrater yazdianus* Rech.f.	Gol-e Arvane Yazdi	leaves	antimicrobial, antiparasitic, antioxidant, anticancer and antidiabetic activities	Yazd	[90]
*Mentha mozaffarianii* Jamzad.	Pooneh-Kooh	aerial parts, leaves, and seeds	stomachache, cramps, chest pain, bronchitis, and colds	Hormozgan, Fars	[91,92]
*Nepeta binalodensis* Jamzad.	Binaludi Pune-sa	aerial parts	headache, migraine, digestive, rheumatism, respiratory disorders, asthma, common cold, colic and cardio-vascular disorders	Khorasan	[25,93,94]
*Nepeta cephalotes* Boiss.	Kopei Pune-sa	aerial parts	diuretic, diaphoretic, antitussive, antispasmodic, anti-asthmatic, febrifuge, emmenagogue and sedative agents	Tehran	[25]
*Nepeta crassifolia* Boiss. & Buhse	Alborzi Pune-sa	aerial parts	cardiovascular complaints such as angina pectoris, cardiac thrombosis, tachycardia, and weakness of the heart	Ardabil, East Azarbaijan	[25,26,95,96]
*Nepeta crispa* Willd.	Mavaj Pune-sa	aerial parts	sedative, relaxant, carminative, tonic for respiratory and nervous disorders	Hamadan, Kermanshah, Chaharmahal va Bakhtiari, Tehran	[25,97]
*Nepeta denudata* Benth.	Oryan Pune-sa	aerial parts	diuretic, diaphoretic, antitussive, antispasmodic, anti-asthmatic, febrifuge, emmenagogue and sedative agents	Tehran	[25]
*Nepeta Depauperata* Benth.	Sabzposhani Pune-sa	aerial parts	diuretic, diaphoretic, antitussive, antispasmodic, anti-asthmatic, febrifuge, emmenagogue and sedative agents	Kerman	[25,98]
*Nepeta dschuparensis* Bornm.	Jupari Pune-sa	aerial parts	diuretic, diaphoretic, antitussive, antispasmodic, anti-asthmatic, febrifuge, emmenagogue and sedative agents	Fars, Kerman	[25,99]
*Nepeta elymaitica* Bornm.	Ilami Pune-sa	aerial parts	diaphoretic, antitussive, antispasmodic, anti-asthmatic, febrifuge, emmenagogue, sedative, and diuretic	Esfahan	[25]
*Nepeta schiraziana* Boiss.	Shirazi Pune-sa	aerial parts	antitussive, antispasmodic, anti-asthmatic, febrifuge, emmenagogue, sedative, diuretic, and diaphoretic	Lorestan, Esfahan, Chaharmahal va Bakhtiari, Fars, Khorasan, Semnan	[25]
*Nepeta glomerulosa* Boiss	Anboh Pune-sa	aerial parts	antispasmodic, anti-asthmatic, febrifuge, emmenagogue, sedative, diuretic, diaphoretic, and antitussive	Mazandaran, Esfahan, Fars, Kerman, Khorasan, Tehran	[25,100]
*Nepeta heliotropifolia* Lam.	Aftab-parasti Pune-sa	aerial parts	anti-asthmatic, febrifuge, emmenagogue, sedative, diuretic, diaphoretic, antitussive, and antispasmodic	Markazi, Qazvin	[25,101]
*Nepeta involucrate* Bornm.	Gariban dar Pune-sa	aerial parts	febrifuge, emmenagogue, sedative, diuretic, diaphoretic, antitussive, antispasmodic, and anti-asthmatic	-	[25]
*Nepeta ispahanica* Boiss.	Esfahani Pune-sa	aerial parts	emmenagogue, sedative, diuretic, diaphoretic, antitussive, antispasmodic, anti-asthmatic, and febrifuge	Esfahan	[25,94]
*Nepeta kotschyi* Boiss.	Kohe Dalv Pune-sa	aerial parts	sedative, diuretic, diaphoretic, antitussive, antispasmodic, anti-asthmatic, febrifuge, and emmenagogue	-	[25]
*Nepeta mentoides* Boiss. & Buhse.	Sabalani Pune-sa	aerial parts	antispasmodic, anti-asthmatic, febrifuge, emmenagogue, sedative, diuretic, diaphoretic, and antitussive	East and West Azarbaijan	[25,102]
*Nepeta persica* Boiss.	Irani Pune-sa	aerial parts	febrifuge, emmenagogue, sedative, diuretic, diaphoretic, antitussive, antispasmodic, and anti-asthmatic	Ardabil, Esfahan	[25,96,103]
*Nepeta rivularis* Bornm.	Juybari Pune-sa	aerial parts	emmenagogue, sedative, diuretic, diaphoretic, antitussive, antispasmodic, anti-asthmatic, and febrifuge	Kerman	[25]
*Nepeta sintenisii* Bornm.	Torkamani Pune-sa	aerial parts	diuretic, diaphoretic, antitussive, antispasmodic, anti-asthmatic, febrifuge, emmenagogue and sedative agents	Mazandaran	[25,96,104]
*Otostegia persica* Boiss.	Golder, Gol-e-kharu	top flowering aerial parts	antispasmodic, antihistaminic, antimalarial, anti-arthritis, diabetes, arthritis, gastric discomfort, headache, rheumatism, sedative activities, regulating blood pressure, and hyperlipidemia.	Fars, Kerman, Sistan va Baluchestan	[105,106,107,108]
*Satureja avromanica* Maroofi.	Marzeh Oramani	aerial parts	antimicrobial, antioxidant, antispasmodic, and anti-diarrheal	Kurdestan	[106,108]
*Satureja Edmondi* Briquet.	Marzeh Edmondi	aerial parts	antimicrobial, antioxidant, antiviral activity (against HIV), and improvement of fertility	Kermanshah, Lorestan, Chaharmahal va Bakhtiari	[109,110]
*Satureja atropatana* Bunge.	Marzeh Azarbaijani	aerial parts	gastroenteritis, upperrespiratory tract infections, urinary tract infections, diarrhea, and wound healing	East and west Azarbaijan	[109,110]
*Satureja bachtiarica* Bunge	Marzeh Bakhtiari	aerial parts	antimicrobial, antioxidant, antispasmodic, anti-diarrheal, and antitumor activities	Kurdestan, Kermanshah, Lorestan, Chaharmahal va Bakhtiari, Fars	[109,110,111,112]
*Satureja intermedia* C. A. Mey.	Marzeh Taleshi	aerial parts	upper respiratory tract infections, urinary tract infections, diarrhea, wounds, and gastroenteritis	Gilan, Ardabil	[109,110]
*Satureja isophylla* Rech. f.	Marzeh Jorbarg	aerial parts	urinary tract infections, diarrhea, wounds, gastroenteritis, and upper respiratory tract infections	Mazandaran	[109,110]
*Satureja kallarica* Jamzad.	Marzeh Kellari	aerial parts	diarrhea, wounds, gastroenteritis, upper respiratory tract infections, and urinary tract infections	Chaharmahal va Bakhtiari	[106]
*Satureja Kermanshahensis* Jamzad.	Marzeh Kermanshahi	aerial parts	wounds, gastroenteritis, upper respiratory tract infections, urinary tract infections, and diarrhea	Kermanshah	[106]
*Satureja khuzistanica* Jamzad.	Marzeh Khuzistani	aerial parts	antifungal, antibacterial, antinociceptive, antioxidant, antidiabetic, antihyperlipidemic, anti-inflammatory, and triglyceride-lowering activities	Khuzestan	[113]
*Satureja rechingeri* Jamzad.	Marzeh rechingeri	aerial parts	antioxidant, antidiabetic, antihyperlipidemic, anti-inflammatory, antifungal, antibacterial, antinociceptive, and triglyceride-lowering activities	Ilam	[103,106,107]
*Satureja sahandica* Bormn.	Marzeh Sahandi	aerial parts	gastroenteritis, upper respiratory tract infections, urinary tract infections, diarrhea, and wounds	East and West Azarbaijan, Kurdestan, Zanjan	[109]
*Stachys acerosa* Boiss.	Sonboleh Kohsari Sonboleh Kharaloud	aerial parts	antispasmodic, diuretic, asthmatic, rheumatic antibacterial, and antioxidant	Kerman, Hamadan, Lorestan, Arak, Esfahan, Kohkilouyeh va Boyer-Ahmad, Chaharmahal va Bakhtiari, Fars	[114,115]
*Stachys asterocalyx* Rech. f.	Sonboleh Shirazi	aerial parts	genital tumors, sclerosis of the spleen, inflammatory tumors, cough, and ulcers	Fars	[116]
*Stachys benthamiana* Boiss.	Sonboleh Sakhreh Zei	aerial parts	antibacterial, antifungal, antioxidant, anxiolytic, anti-inflammatory, hypotensive, and anti-nephritic activities	Fars, Chaharmahal va Bakhtiari, Esfahan	[114,115]
*Stachys laxa* Boiss. & Buhse.	Sonboleh Damavandi	aerial parts	anticancer, antibacterial, antioxidant, anti-inflammatory, anti-nephritic, anti-anxiety	Golestan, Mazandaran, Semnan, Tehran	[117,118]
*Stachys obtusicrena* Boiss.	Sonboleh Kongerei	aerial parts	genital tumors, sclerosis of the spleen, inflammatory tumors, cough, ulcers, and infected wounds	East and West Azarbaijan, Yazd, Gilan	[116]
*Stachys pilifera* Benth.	Sonboleh Modar	aerial parts	asthma, rheumatoid arthritis, antioxidant, antimicrobial, anti-inflammatory, and antitumor	Yasuj	[119,120]
*Thymus carmanicus* Jalas.	Avishan-e-kermani	aerial parts	antispasmodic, antimycotic, mammalian age-delaying properties, bactericides, antiseptics, antioxidants, and anthelmintic properties	Kerman	[112,121]
*Thymus deanensis* Celak.	Avishan-e-denaee	aerial parts	antibacterial	Hamadan, Azarbaijan, Chaharmahal va Bakhtiari, Kurdestan, Hamadan, Kermanshah, Esfahan, Tehran, Fars, Kerman	[112,121,122,123,124,125]
*Thymus eriocalyx* (Ronninger.) Jalas.	Avishan-e-Korkalod	aerial parts	gastrointestinal disturbances	Lorestan	[124,125]
*Thymus fallax* Fisch. & C. A. Mey.	Avishan-e-Anatoli	aerial parts	antibacterial, antifungal, antiviral, antiparasitic, spasmolytic, and antioxidant	Hamadan, Tehran	[126,127]
*Thymus kotschyanus* Boiss. & Hohen.	Avishan	aerial parts	gastrointestinal disturbances, anthelmintic, antioxidant, strongly antiseptic, antispasmodic, carminative, deodorant, diaphoretic, disinfectant, expectorant, sedative, and tonic	Mazandaran, Gilan, East, and West Azarbaijan, Tehran, Kurdestan, Yazd	[121,124,128,129]
*Thymus persicus* (Ronniger ex Rech. f.) Jalas.	Avishan-e-Irani	aerial parts	anti-inflammatory, hepatoprotective, antitumor, anti-HIV, antimicrobial, antifungal, anti-ulcer, gastroprotective, hypoglycemic, and antihyperlipidemic	East and West Azarbaijan	[125,130]
*Thymus pubescens* Boiss. & Kotschy ex Celak.	Avishan-e-korkaloud	aerial parts	tonic, carminative, digestive, antispasmodic, anti-inflammatory, and expectorant	East and West Azarbaijan, Mazandaran, Tehran	[121]
*Thymus trautvetteri* Klokov & Desj.- Shost.	Avishan-e-Taleshi	aerial parts	tonic and herbal tea, flavoring agents (condiment and spice), antiseptic, antitussive, and carminative, as well as treating colds	Mazandaran	[131]
*Zataria multiflora* Boiss.	Avishane Shirazi	aerial parts	immunostimulant, antinociceptive, anti-inflammatory, antioxidant, antibacterial, antiviral, antiparasitic, and antifungal	Kerman	[112,132,133,134]
*Zhumeria majdae* Rech. f. & Wendelbo.	Mohrekhosh,	leaves	stomach tonic, antiseptic anti-nociceptive, and anti-inflammatory	Hormozgan	[135,136]
*Ziziphora capitata* Linnaeus.	kakuti-e Sarsan	aerial parts	sedative, stomach tonic, flatulence, common cold, diarrhea, expectorant, coughing, antiseptic, migraine, and carminative	Kurdestan	[90,137]
*Ziziphora clinopodioides* Lam.	kakuti-e kuhi	aerial parts	hypertension, sedative, stomach, tonic, heart disorders, common cold, inflammation, depression, diarrhea, expectorant, coughing, antiseptic, migraine, carminative, and wound healing	Mazandaran, Semnan, Tehran, Kerman, Chaharmahal va Bakhtiari, Esfahan	[123,137]
*Ziziphora persica* Bunge.	kakuti-e Irani	aerial parts	antimicrobial	East and West Azarbaijan	[137]
*Ziziphora tenuior* Linnaeus.	Kakuti	aerial parts	antimicrobial	Chaharmahal va Bakhtiari	[112,137]
Liliaceae (1)					
*Lilium ledebouri* Boiss.	Susan-e Chelcheragh	corm, flower	burns, injuries, inflammation, and uterus disorders	Mazandaran, Gilan	[138]
Rosaceae (3)					
*Amygdalus elaeagnifolia* Spach.	Badame- Kermani	fruit, seed	healing effects on skin damages caused by radiotherapy, anxiolytic properties, have a decreasing effect on anxiety and stress	Lorestan, Arak, Kohkilouyeh va Boyer-Ahmad, Chaharmahal va Bakhtiari, Fars, Kerman	[139]
*Amygdalus scoparia* Spach.	Badame Kohi	aerial parts	diabetes mellitus	Fars	[139]
*Amygdalus lycioides* Spach.	Badame Vahshi	aerial parts	hyperlipidemia, hypoglycemia	East and West Azarbaijan, Lorestan, Arak, Esfahan, Yazd, Kerman, Tehran, Hormozgan	[139]
Scrophulariaceae (1)					
*Verbascum sublobatum* Murb.	Gol-e Mahor	leaf	antioxidant	Golestan, Mazandaran, Tehran	[140,141]

### 3.2. Endemic MAPs in Iran and the Plant Parts in Use

**Figure 3:**
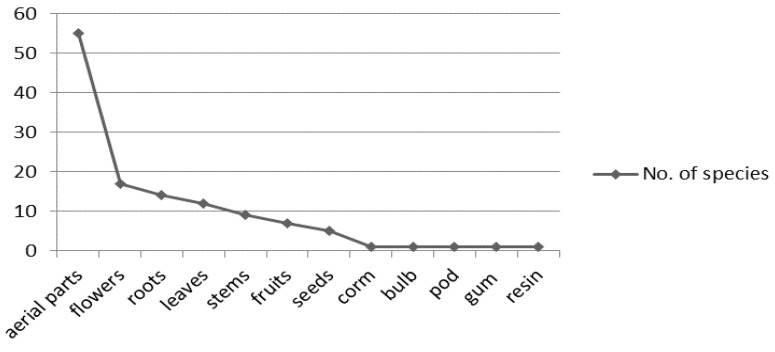
The plant parts of the Iranian endemic medicinal and aromatic plants in common use for curing the diverse maladies.

Endemic medicinal plants are used for the relief of many disease conditions, namely respiratory system diseases, digestive system disorders, and muscular-skeletal system problems. The plant parts used for medicine (based on the species frequency) are aerial parts (55 spp.), flowers (17 spp.), roots (14 spp.), leaves (12 spp.), stems (9 spp.), fruits (7 spp.), seeds (5 spp.), corms (1 spp.), bulbs (1 spp.), pods (1 spp.), gums (1 spp.), resins (1 spp.), and manna (1 spp.) (Figure 3). The aerial parts are used in 55 out of 100 species. A total of 17 species are used for their flowers, which is followed by roots with 14 species (Table 1 and Figure 3). In many cases, more than one organ of the same species is used in the treatment of different maladies (Table 1).

### 3.3. Endemic MAPs Richness across Iran

**Figure 4:**
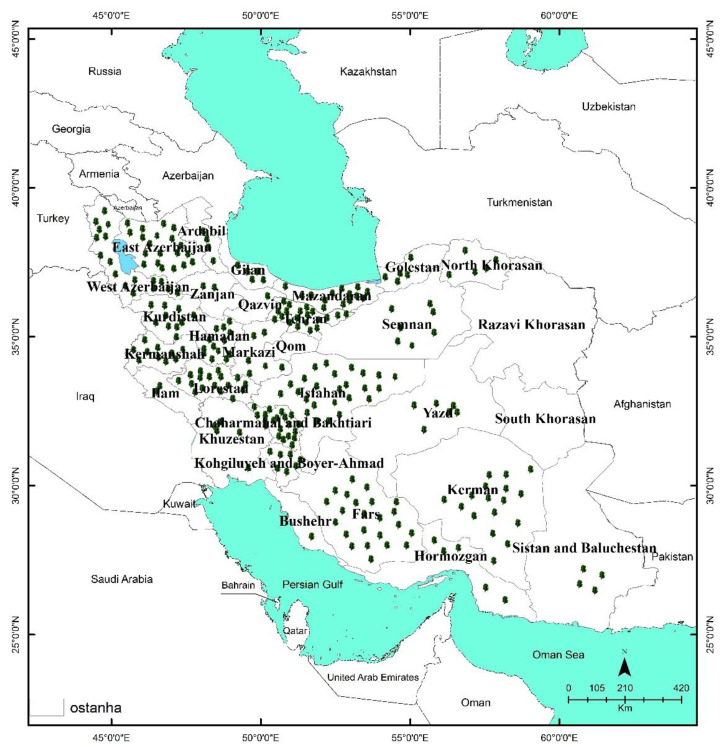
The distribution pattern of Iranian endemic medicinal and aromatic plants across the provinces.

**Figure 5:**
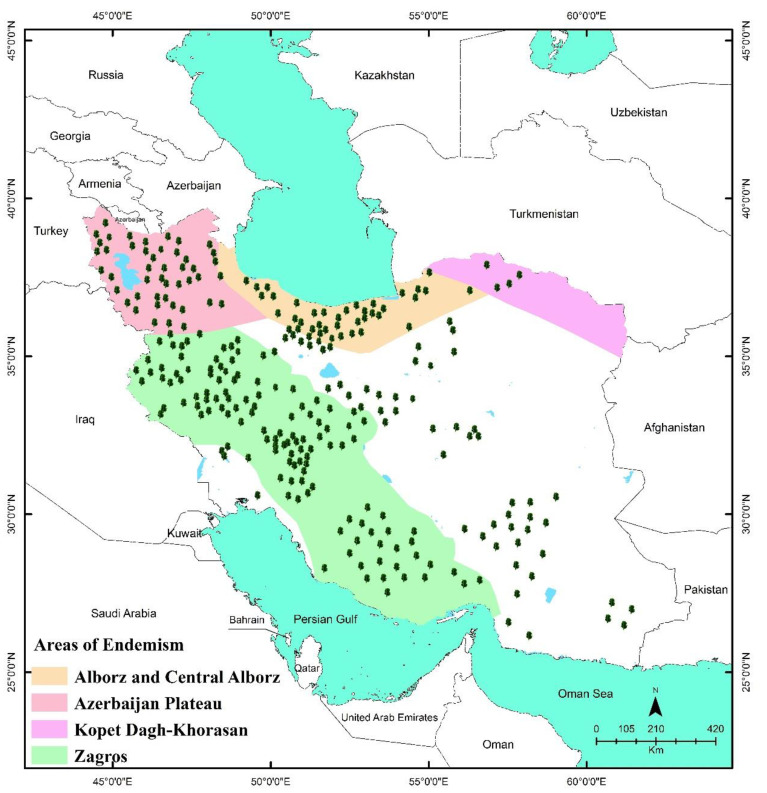
Distribution patterns of the endemic medicinal and aromatic plants of Iranian flora by endemism area.

**Figure 6:**
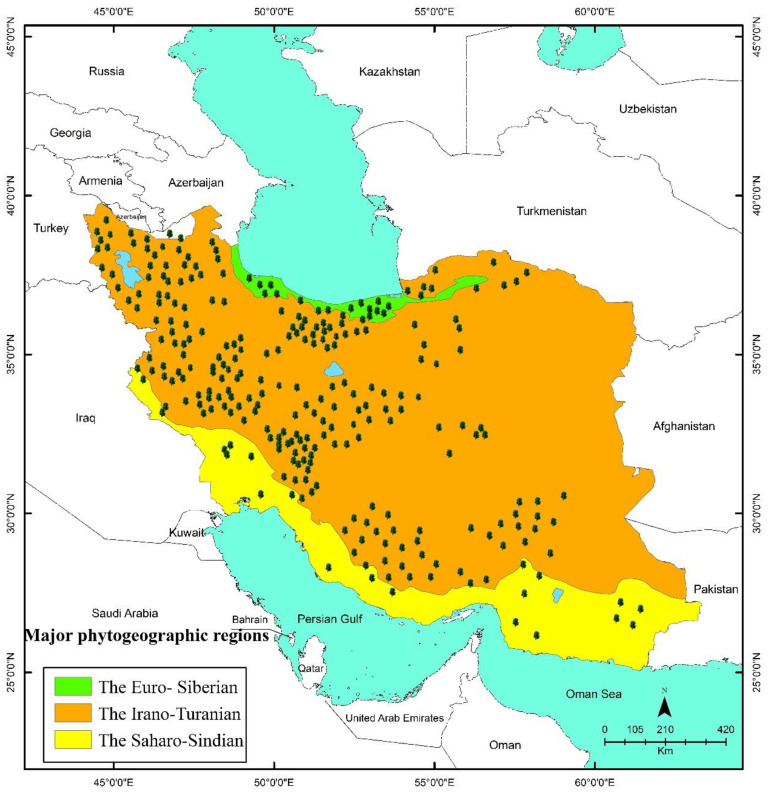
Topogeographic map of Iran indicating phytogeographical regions and distribution of endemic medicinal and aromatic plants.

The distribution of Iranian endemic MAPs is shown in Figure 4, Figure 5 and Figure 6. As seen on the maps, the endemic MAPs of Iranian flora are distributed in nearly all parts of the country. Most of these valuable plants flourish in small populations in mountainous habitats. In Southwest Asia in general and in Iran in particular, mountains have the dominant role in the development of endemic species [14].

Endemic richness in Iran is significantly related to the topography and the climatic conditions of the regions and numerous mountain ranges [18]. There are many mountain peaks in Iran with an elevation higher than 4000 m [15], and signficant richness was reported in high mountains (Sabalan Mts., Sahand Mts., Talysh Mts., Shahu Mts., Alvand., Oshtorankuh Mts., Zardkuh Mts., Dena Mts., Alvand Mts., Karkas Mts., Binalood Mts., Shirkuh Mts., and Hezar-Lalezar Mts.) [1].

Most of the published information on the number of endemic MAPs of Iran is based on the political units, such as provinces. The number of endemic MAPs varies greatly among 31 provinces (from 0 in Qom to 25 in Esfahan) (Figure 4). Most of them are distributed in the main mountain ranges of Esfahan, Kerman, Fars, Tehran, Chaharmahal va Bakhtiari, East Azarbaijan, Lorestan, West Azarbaijan, Hamadan, and Mazandaran provinces, respectively (Figure 4).

Noroozi et al. (2018) reported five AEs from Iran, (i.e., Zagros, Azarbaijan, Kopet Dagh-Khorassan, Alborz, and Central Alborz) (Figure 5). The ten provinces mentioned above are predominantly located in these five AEs (Figure 5), which are in the Iran-o-Turanian region, one of the three major phytogeographic regions in Iran (i.e., the Saharo-Sindian, the Iran-o-Turanian, and the Euro-Siberian) (Figure 6). The Iran-o-Turanian region composes the highest percentage (68.29%) of endemic MAPs in Iran (Figure 6) and is considered as an essential area of endemism in Asia [1,12,15].

Among AEs of Iran, Zagros was found to host the maximum number of endemic MAPs with 125 species, followed by Azarbaijan (46 species), Alborz, and Central Alborz (40 species), and Kopet Dagh-Khorassan (4 species) (Figure 5). The least number of endemic MAPs was found in Kopet Dagh-Khorassan with only four species. The low number of endemic MAPs in Kopet Dagh-Khorassan results from the limited size of the area. The mountainous areas of Kopet Dagh-Khorassan do not expand beyond Iran except for a small section in the north [1].

**Figure 7:**
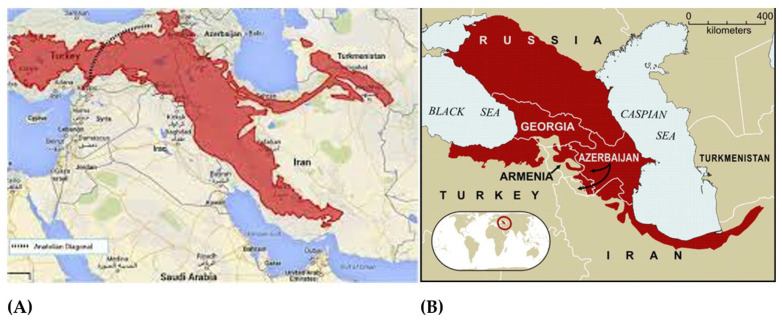
Two biodiversity hotspots regions of Iran (adopted from https://biodiversity.doe.ir/portal/home/?778961/ (accessed on 26 January 2022). Note: (**A**) shows Irano-Anatolian biodiversity hotspot, and (**B**) shows the Caucasus biodiversity hotspot.

Five AEs (Alborz, Central Alborz, Zagros, Azarbaijan, and Kopet Dagh-Khorassan) and three phytogeographic regions (the Iran-o-Turanian, the Saharo-Sindian, and the Euro-Siberian) in Iran cover parts of two global BHs (i.e., Irano-Anatolian and Caucasus), and it is estimated that 97% of the endemic vascular plant species of this country are limited to these sectors [12]. The Caucasus hotspot includes Georgia, Azarbaijan, and a small portion of northern Iran, and the Iran-Anatolian hotspot includes significant parts of northern and western Iran, central and eastern Turkey, a small portion of southern Georgia, the Nakhchivan province of the country of Azerbaijan, much of Armenia, northeastern Iraq, and the northern Kopet Dagh range in Turkmenistan (Figure 7).

Our findings on the distribution pattern of endemic MAPs in Iran is similar to the results of a study at the University of Vienna about the distribution of the Asteraceae family as a model group in Iran [14], a study at the University of Tehran about the distribution of Iranian trees and shrubs [19], and a study about the biodiversity and floristic endemism of *Fritillaria* spp. in Iran [13]. Our results verify the vast distribution pattern for a large number of species across the country, and even clarify the diversification of many unconsidered species with medicinal values that have not been the focus of former studies.

### 3.4. Relationship between the Climate and Richness of Endemic MAPs of Iran

**Figure 8:**
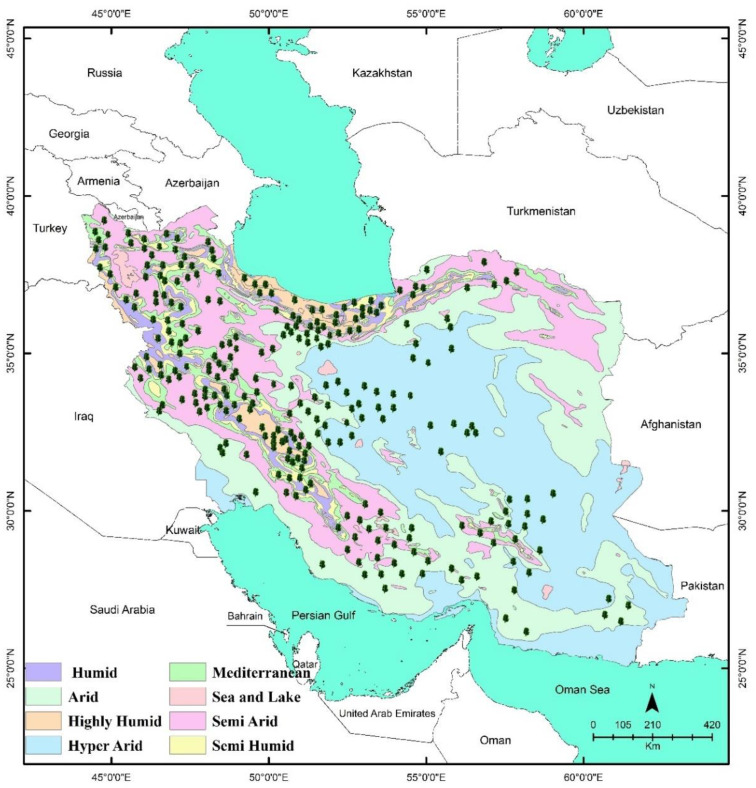
Climate map of Iran showing the location of endemic medicinal and aromatic plants.

Iran, as a vast country with 31 political provinces, has significant cross-sectional variation in the climate types and is characterized by different climates ranging from arid to semi-arid mountain ranges. Various climates of Iran include arid, semi-arid, hyper-arid, humid, semi-humid, highly-humid, and Mediterranean [17] (Figure 8). Thus, due to the particular distribution of endemic MAPs and different climates in Iran, we investigated how endemic MAPs correlate with the different climate conditions in this country.

As shown in Figure 8, the climate of one province is mainly different from that of another. The provinces we studied were found to host a diverse range of endemic MAPs, dominantly belonging to five different climate conditions, i.e., semi-arid, humid, semi-humid, highly-humid, and Mediterranean. The maximum number of species (86 spp.) were found in the semi-arid climate, followed by the humid (28 spp.), highly-humid (27 spp.), semi-humid (14 spp.), and the Mediterranean (12 spp.) climates (Figure 8). Overlapping of the species within the areas has been observed (Figure 8).

Like all other species of the biosphere, MAPs have no exemption from the effects of climate change [27], especially some MAPs that are endemic to the geographic regions which are more at risk and vulnerable to climate changes [28]. Climate change is attributable directly or indirectly to the human enterprises that alter atmospheric composition [28]. There is a high risk of mass extinction of biodiversity as the planet warms and the climate changes, and Iran is also impacted by climate change, especially due to the presence of mountains and near-desert areas. For example, some cold-adapted MAPs in mountainous hillsides in Iran have begun to gradually migrate higher up mountain summits, a phenomenon correlated with climate warming [15]. Eventually, this migration of MAPs may cause them to face extinction [15]. Higher temperatures and lower water availability can cause climate changes that likely have a significant impact on MAPs growth in the near future [29].

Climate change will alter the environmental conditions for MAPs, especially in arid and semi-arid regions. In other words, some regions in the “Humid” class may be turned into the “Arid” class, and some regions that are currently “Semi-Arid” may be turned into the “Hyper-Arid” class regarding the climatic change [17].

Some MAPs are drought-tolerant, and the stress may cause increases in the concentration of their secondary metabolites (either by decreasing biomass or by increasing the actual production of the metabolites). For other species, however, relationships with specific pollinators may be disrupted by the phenological alterations arising from climate change [27].

## 4. Need for Conservation

The distribution of the MAPs on the Earth is not uniform and differs in different geographical regions. Regardless, they serve a considerable role in the health care of people across the world. As per the data available, more than 75% of the world’s population relies mainly on medicinal plants and herbal medicines for their health care needs [6,8,9].

The geographic distribution and biological attributes of these kinds of plants must be known to guide conservation programs and better manage our use of the biosphere [6]. Human management must be able to balance the competing demands of obtaining the greatest resources for the present generation while preserving the potential for future generations. In the preservation approach, all species are not of equal significance, and setting priorities is the most important step in the conservation programs [4]. In this context, endemic MAPs with limited distribution are of greater importance than exotic species with a wide distribution.

The geographic distribution of endemic MAPs must be known to guide the conservation proceedings, e.g., to assess whether species protection should take place in nature or a greenhouse [6]. Two sets of suggestions relating to the conservation of endemic MAPs have been developed as follows: in situ (protection of species in their natural environment) and ex situ (protection of species outside their natural habitat) [6,9]. Both conservation strategies (i.e., in situ and ex situ) and also resource management (e.g., good agricultural practices and sustainable use solutions) should be adequately taken into account for the sustainable use of MAPs resources [6].

Nowadays, biotechnological tools, such as micropropagation, tissue culture, synthetic seed technology, and molecular markers, provide new and complementary options for plant conservation, including short-, medium-, and long-term strategies, and their application for plant species conservation has considerably increased [30]. Indeed, no conservation strategy alone may be sufficient to prevent species from extinction. Therefore, it is important to combine conservation strategies (in situ, ex situ, biotechnological tools, and so on) that will complement each other in the effort to preserve a given species.

## 5. Perspectives for Conservation and Habitat Management of MAPs

In summary, while MAPs have contributed to the healthcare systems and economy of rural populations, the following critical issues should be addressed:

1. The national and international demand for MAPs in Iran is increasing, which creates tremendous pressure on natural habitats. There is no formal harvesting system, so little is known about which plants are being harvested, from where, and in what quantity. There is a clear need for a system of monitoring and tracking wild harvesting, specifically in the five AEs.
2. To reduce the harsh harvesting pressure on wild populations and to conserve the vulnerable species, novel technologies should be introduced to improve the culture, harvesting, and drying of the MAPs, especially the value-added endemic medicinal and aromatic plants.
3. The programmed utilization management of the endemic MAPs can generate more income for the farmers and local harvesters and, therefore, protects the environment and MAPs populations from the excessive harvests and possible extinction.
4. In addition to the efforts for mitigating the extinction of endemic MAPs, the preservation of traditional knowledge is a component of conservation. Folk understanding of medicinal and aromatic plants used by the inhabitants of Iran should be recorded, particularly in the rural areas of the country, where there is no or limited access to hospitals, drugstores, and health experts.
5. Most of the published information on the number of endemic MAPs of Iran is related to the political provinces. Many large provinces in the arid or hyper-arid regions are relatively poor in endemic MAPs, while many provinces in the humid, highly-humid, semi-humid, semi-arid, and Mediterranean regions are extraordinarily rich in endemic MAPs. Consequently, studying the environmental potentials and risks of these provinces would protect the majority of the endemic plants from excessive harvest regimes and the possible extinction.
6. Although, species conservation, especially that of the endangered species, may be more effective through natural habitat (in situ conservation) inspections and environmental managements; the ex-situ techniques also can be used to complement the in-situ methods. Biotechnological methods such as micropropagation, tissue culture, synthetic seed technology, and molecular marker approaches can be used to amend the product and alter the efficacy of medicinal and aromatic plants.
7. Supplementary studies can be carried out in other fields such as phylogenetic diversity or DNA barcoding to emphasize the importance of the endemic MAPs, the AEs, and the conservation programs.
8. 16.7% of Earth’s land surface is home to 77% of all endemic plant species representing 36 BHs in the world, and Iran holds two of the biodiversity hotspots. So, to conserve Iran’s biodiversity, the Iranian government should launch projects to conduct biodiversity investigation across the country, especially in endemic MAPs and specifically in Tehran, Esfahan, Kerman, Fars, Chaharmahal va Bakhtiari, East Azrbaijan, Lorestan, West Azarbaijan, Hamadan, and Mazandaran provinces, to more thoroughly understand their distribution, abundance, and ecology for the long-term and sustainable production of major MAPs.
9. By describing the geographical distribution and ecological aspects of the endemic MAPs along with a concentrated insight into their biodiversity concerning their taxonomic status, a more obvious understanding of the essential elements of conservation strategies can be supplied for all the involved preservationists, government sectors, and NGOs.

## 6. Conclusions

Iran is a country of diverse landforms, climates, and species of MAPs (2300 out of 8200 species). Of the 36 BHs recognized in the world, Iran has two significant hotspots—the Irano-Anatolian and the Caucasus. Reviewing the phytogeographical distribution pattern of MAPs reveals that the Iran-o-Turanian region is the main center of diversity for the Iranian endemic MAPs. Considering the total number of AEs recognized in Iran (Alborz and Central Alborz, Zagros, Azarbaijan, and Kopet Dagh-Khorassan), the number of endemic MAPs will be interesting. Our data show the density of endemism in Esfahan, Kerman, Fars, Tehran, Chaharmahal va Bakhtiari, East-Azarbaijan, Lorestan, West-Azarbaijan, Hamadan, and Mazandaran provinces is higher than in the other provinces of Iran. The dominant MAPs diversity harboring localities of Iran were found to host a diverse range of endemic MAPs (100 species of medicinal and aromatic importance, from 10 families and 30 genera, with potential uses for therapeutic purposes). Although all of Iran is essential for conservation, those areas rich in endemic MAPs that are prone to climate change, are relatively more significant to consider their diversity inspection and further conservation programs. This review article provides the policy-makers baseline data to make suitable decisions for the conservation of endemic medicinal and aromatic plants at the national and provincial levels. With an increasing world population and climate change, the identification of all BHs at a finer scale and identifying AEs of every country are essential elements for the execution of global conservation management programs.

## Figures and Tables

**Table 2:** A list of some neglected endemic MAPs of Iranian flora.

Apiaceae	*Ferula*	*F. macrocolea.*	*F. stenocarpa*	
		*F. microcolea*	*F. behboudiana*	
	*Ferulago*	*F. phialocarpa*		
	*Heracleum*	*H. nephrophyllum*		
	*Pimpinella*	*P. tragioides*		
	*Eryngium*	*E. bungei*		
	*Anthemis*	*A. austroiranica.*	*A. kermanensis*	*A. odontostephana*
	*Echinops*	*E. aucheri*	*E. elymaticus*	*E. Iranshahrii*
		*E. lalesarensis*	*E. macrophyllus*	
	*Helicrysum*	*H. oligocephalum*		
	*Scorzonera*	*S. subaphylla*		
	*Hertia*	*H. angustifolia*		
Boraginaceae	*Onosma*	*O. asperimum*		
Brassicaceae	*Isatis*	*I. pachycarpa*		
Caryophyllaceae	*Dianthus*	*D. macranthoides*		
Fabaceae	*Astragalus*	*A. camptoceras*	*A. globiflorus*	*A. ovinus*
		*A. effusus*	*A. glaucacanthos*	*A. sieversianus*
		*A. ophiocarpus*	*A. crenatus*	*A. tribuloides*
		*A. mucronifolius*	*A. jolderensis*	*A. verus*
	*Hedysarum*	*H. persicum*		
Hypericaceae	*Hypericum*	*H. rechingeri*		
Lamiaceae	*Nepeta*	*N. adenoclada*	*N. gloecocephala.*	*N. mirzayani*
		*N. allotria*	*N. hymenodonta*	*N. oxydonta*
		*N. archibaldii*	*N. iranshahrii*	*N. pogonosperma*
		*N. assurgens*	*N. koeieana*	*N. prostara*
		*N. assadii*	*N. chinophila*	*N. racemose*
		*N. bakhtiarica.*	*N. lasiocephala*	*N. scrophularioides*
		*N. eremokosmos*	*N. laxiflora*	*N. sessilifolia*
		*N. gedrosiaca*	*N. makuensis*	*N. straussii*
	*Thymus*	*T. fedtschenkoi*	*T. migricus*	
	*Hymenocrater*	*H. incanus*	*H. platystegius*	
	*Stachys*	*S. ixodes*		
Malvaceae	*Alcea*	*A. koelzii*		
Liliaceae	*Fritillaria*	*F. kotschyana*	*F. zagrica*	
Linnaceae	*Linum*	*L. persicum*		
Polygonaceae	*Polygonum*	*P. aridum*		
Ranunculaceae	*Clematis*	*C. ispahanica*		
Scrophulariaceae	*Scrophularia*	*S. farinosa*		
	*Verbascum*	*V. gabrielae*		
Xanthorrhoeaceae	*Eremurus*	*E. persicus*		
	*Rheum*	*R. persicum*		
	*Rumex*	*R. crispus*		

## Data Availability

All-new research data were presented in this contribution.
